# Surgery of the Peritoneum

**Published:** 1905-08-26

**Authors:** 


### SURGERY OF THE PERITONEUM.

**Peritonitis.**—Dudgeon and Sargent[1] have made systematic examinations of the peritoneum in 270 cases of operation, and their researches into the bacteriology of peritonitis have been embodied in the Erasmus Wilson Lectures. In gastric and duodenal perforations, in intra-peritoneal hæmorrhage, and in pelvic inflammations the organisms are, in the great majority of cases, of a mild type. In intestinal necrosis, on the other hand, it is the virulent colon bacillus which is by far the most common organism. The streptococcus pyogenes and the bacillus pyocyaneus are so uncommon and so uniformly fatal, whatever the treatment adopted, that it would be wise to treat all cases of peritonitis arising from intestinal necrosis as cases of colon bacillus peritonitis. It is undoubtedly true that peritonitis of bacterial origin can subside without operative interference. But such terminations are not events to be reckoned upon, and the earlier operation is undertaken the less likely is the peritoneal infection to reach a desperate stage. The extent of the operation depends upon the bacteriology of the case. In streptococcic peritonitis, which is the most acute and fatal form we know, it is doubtful whether any surgical procedure offers a prospect of success, but if there is any chance it must lie in the most thorough possible washing. In the vast majority of cases, however, it is the colon bacillus which is the chief infective agent, and here limited operations should be the rule, because of the mechanical impossibility of cleansing the peritoneum, the risk of washing away the phagocytes in the exudate beyond the area of most intense inflammation, and the danger of spreading infection over unaffected regions. It is probable that dry sponging is better than local irrigation, because fluid poured into the abdomen is likely to carry infection beyond the area already involved. If those remote regions are already affected, then no amount of washing is likely to be of much avail. In the case of mild infections, particularly those due to gastric perforations and intraperitoneal hæmorrhage, the washing should be thorough, with a view to remove the foreign material present, such as the contents of the stomach or blood clot. In mild diffuse infections, such as gastric perforations, intraperitoneal hæmorrhage, and the early stages of the peritonitis of obstruction, the organisms are comparatively harmless, and can be safely dealt with by the peritoneum. It is the rule in such cases where the abdomen has been closed without drainage for the wound to heal by primary union. In severe diffuse infections on the other hand, there may be said to be more ground for drainage, although one sees many cases recover without even suppuration of the abdominal wall. In many cases of local abscess there is a definite cavity which for practical purposes is extra-peritoneal, and which can be drained. When there is a sticky exudate, drainage is greatly interfered with. The teachings of bacteriology harmonise with those of clinical experience in pointing to purgatives and not to opiates as the right drugs to be given in peritonitis. Serum treatment gives some promise of utility, but the indiscriminate use of antitoxic sera in peritonitis, without regard to the bacteriology of the individual case, is to be deprecated as being as useless as it is unscientific. Matthews[2] considers that pneumococcus peritonitis is three times as frequent in children as in adults. The most characteristic clinical picture of this form of peritonitis is seen in the apparently primary cases, and three stages may readily be distinguished: (1) Sudden onset with high fever, vomiting for a day or two, tenderness, and distension. There is little muscular rigidity, and the pain and distension are not so marked as in the other types of peritonitis. (2) After a few days vomiting ceases; temperature falls often to normal. Diarrhœa, often present in the first stage of the disease, is the rule in the second stage. The amelioration of symptoms is pronounced, appetite returns, and the child looks better. (3) Then with the increase of exudate there appears a tense cystic mass in the hypogastrium, temperature rises, and shows marked morning and evening remissions and exacerbations. There is cachexia and weakness, and the case terminates in from one to four months in death from exhaustion or in recovery following operation or perforation at the umbilicus. There are two types of the disease—the diffuse, which may be expected to result fatally unless future experience shows that cases can be headed off by early operation; and the localised, which usually results fatally unless operation is undertaken. Eighty per cent. of the localised form may be expected to recover after operation. The late stages of the disease simulate tuberculous peritonitis. In the second stage, with diarrhœa and slight distension, the picture is not unlike that of typhoid. Other cases resemble appendicitis and in some cases the appendix has been the source of infection. Haegler[3] considers that a free serous exudation of the peritoneum should be regarded as an early symptom of perforative peritonitis. As the result of animal experimentation he has learned that the quantity of the exudate stands in direct proportion to the amount or virulence of the germ material or its toxin that has been introduced. The peritoneum reacts to all irritation very rapidly, and this reaction is shown by the excretion of a serous exudate, and it may be stated that absorption occurs in an almost similar length of time. This exudation is a movement of defence on the part of the peritoneum against irritation or infection. In those cases of local inflammation of the intestinal wall the presence of free fluid in the peritoneal cavity should be considered as being unfavourable. It shows that there is no tendency towards a restriction of the area of inflammation, for as soon as an attempt is made to restrict this inflammation adhesions rapidly develop. Borchardt[4] considers it possible to increase the resistance of the peritoneum to infection, and in this way to lessen the danger of abdominal operation. The intraperitoneal injection of physiological salt solution generally effects this object better than injections of nucleic acid or horse serum. The greatest immunity appears to be about 48 hours after the injection and the immunity thus acquired lasts about four days.

[1] Lancet, Feb. 25, 1905, p. 473; March 4, 1905, p. 548; March 11, 1905, p. 617. [2] Annals of Surg., Nov. 1904, p. 698. [3] Amer. Jour. Med. Sci., Nov. 1904, p. 926. [4] Med. Chron., Jan. 1905, p. 252.

